# The role of epigenetic modifications in cancer-associated fibroblasts

**DOI:** 10.3389/fmed.2026.1780734

**Published:** 2026-04-24

**Authors:** Binghang Yan, Chengxiao Yang, Xinyuan Liu, Qinwen Zuo, Jing Wang, Wenbo Yang, Hongze Chen

**Affiliations:** 1 Department of Hepatobiliary and Pancreas Surgery, The First Affiliated Hospital of Harbin Medical University, Harbin, China; 2 Key Laboratory of Hepatosplenic Surgery, Ministry of Education, The First Affiliated Hospital of Harbin Medical University, Harbin, China; 3 Department of Pancreatic and Biliary Surgery, The First Affiliated Hospital of Harbin Medical University, Harbin, China

**Keywords:** Cancer-associated fibroblasts (CAFs), epigenetic modification, tumor matrix, tumor microenvironment, tumor stroma component

## Abstract

Cancer-associated fibroblasts (CAFs) constitute a heterogeneous population of stromal cells whose tumor-modulating activities are highly context-dependent. Unlike cancer cells, CAFs rarely harbor stable genetic mutations; instead, their phenotypic plasticity is largely driven by reversible epigenetic reprogramming. Within the tumor microenvironment (TME), CAFs function as key regulators by secreting extracellular matrix (ECM) components, paracrine growth factors, cytokines, and metabolic intermediates that collectively promote tumor growth, invasion, and therapeutic resistance. Recent advances in cancer biology have shifted attention from solely cataloging coding-sequence mutations toward understanding the epigenetic mechanisms that shape CAF identity and function. Major epigenetic regulatory mechanisms include DNA methylation, histone modifications, and RNA modifications, which dynamically regulate chromatin structure and gene expression without altering the underlying DNA sequence. Emerging evidence indicates that the activation and functional heterogeneity of CAFs are predominantly governed by these epigenetic alterations rather than by permanent genetic changes. Epigenetic reprogramming enables CAFs to acquire diverse tumor-promoting properties, including remodeling of the extracellular matrix, modulation of intercellular signaling pathways, and secretion of cytokines that influence gene expression in neighboring cancer and immune cells. These processes ultimately contribute to tumor initiation, progression, metastasis, and resistance to therapy. This review summarizes recent advances in understanding how epigenetic modifications regulate CAF activation and function within the tumor microenvironment. In addition, we discuss the potential of targeting CAF-associated epigenetic pathways as a promising therapeutic strategy for cancer treatment.

## Background

The tumor microenvironment (TME) is a complex ecosystem composed of the extracellular matrix, vascular and lymphatic networks, adipocytes, immune cells, and cancer-associated fibroblasts, all of them collectively induce tumor initiation and metastatic dissemination (1). As pivotal stromal components, CAFs—also referred to as activated fibroblasts—play essential roles in tumor progression. CAF-derived exosomes facilitate tumor growth and contribute to therapeutic resistance.

Epigenetic modification refers to heritable changes in gene function that occur without mutation in the DNA sequence itself, primarily mediated through chemical modifications of DNA, RNA, or relative proteins (2). Broadly defined, epigenetic mechanisms include DNA methylation, histone post-translational modifications, ATP-dependent chromatin remodeling, regulatory non-coding RNAs, and RNA chemical modifications. These mechanisms regulate cellular function and tissue architecture through targeted molecular alterations.

Epigenetic regulation plays crucial roles in diverse biological processes, including embryonic development, tissue repair, aging, and physiological responses to environmental stimuli (3). Dysregulation of epigenetic processes will induce a hallmark of numerous human diseases, particularly tumor (4).

Accumulating evidence shows that epigenetic modification reshapes both the transcriptome of malignant cells and the functional identity of stromal cells, particularly CAFs as tumors evolving (5). Epigenetic alterations promote carcinogenesis by enhancing cell proliferation, facilitating invasion and metastasis, and metabolism reprogramming (6). Furthermore, epigenetic modification regulate extracellular matrix deposition and tissue stiffness, contributing to the formation of a fibrotic and tumor-supportive stromal environment.

### Origin and heterogeneity of CAFs

Fibroblasts were first described by Virchow in 1858. They originate from mesenchymal cells and are widely distributed throughout connective tissues. Fibroblasts play essential roles in maintaining extracellular matrix homeostasis, preserving tissue architecture, promoting wound repair, and regulating immune responses (7).

In physiological conditions, fibroblasts keep in a quiescent state characterized by minimal intercellular communication, limited collagen synthesis, low cytokine secretion, and restricted proliferative activity (8). However, during wound healing, inflammation, or fibrotic processes, fibroblasts can transform into myofibroblasts characterized by increased expression of α-smooth muscle actin (α-SMA) (4). Activated fibroblasts exhibit enhanced contractility and an increased capacity for extracellular matrix production and remodeling, thereby promoting tissue repair and structure (9). Following the completion of tissue repair, these cells typically undergo apoptosis or revert to a quiescent fibroblast phenotype (10).

In the tumor microenvironment, CAFs represent the dominant stromal cell population. CAFs originate from various cells, including resident tissue fibroblasts, bone marrow–derived mesenchymal stem cells, epithelial cells undergoing epithelial–mesenchymal transition (EMT), endothelial cells undergoing endothelial–mesenchymal transition (EndoMT), pericytes, adipocytes, smooth muscle cells, and macrophages (11). During tumor progression, numerous signals—including TGF-β, LIF, IL-1, TNF-α, IL-6, activation of the JAK/STAT3 pathway, hypoxia-induced HIF-1 stabilization, Notch signaling, reactive oxygen species, and cytotoxic therapies—can induce fibroblast activation and their transformation into CAFs (12). These factors can promote extracellular matrix deposition, fibrotic remodeling, angiogenesis, and the recruitment of tumor-associated immune cells, creating a microenvironment supporting tumor growth and invasion.

**Figure 1:**
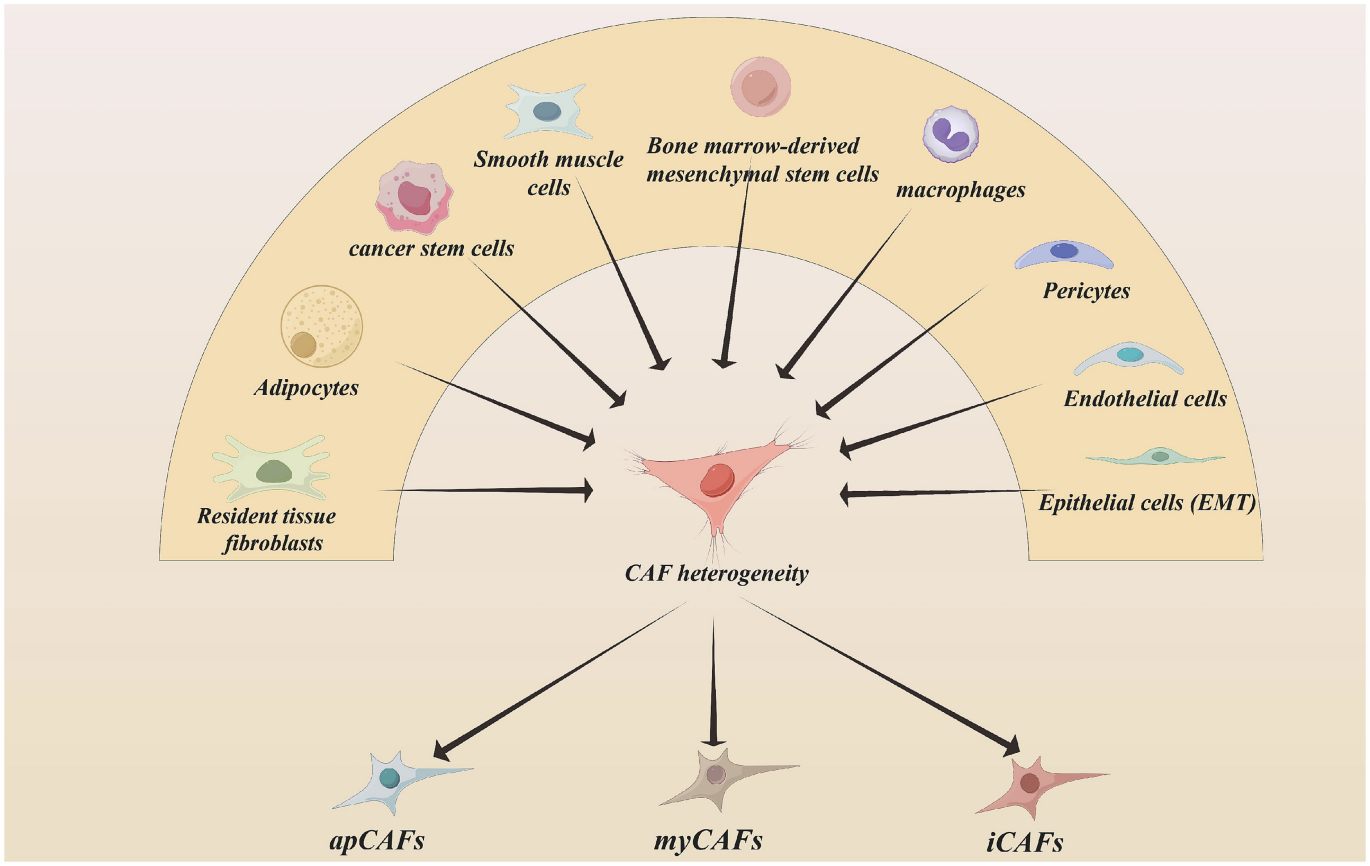
The origin of CAFs. The origination of CAFs is presented in this figure, including resident tissue fibroblasts, adipocytes, cancer stem cells, smooth muscle cells, bone marrow-derived mesenchymal stem cells, macrophages, pericytes, endothelial cells, epithelial cells. The whole CAFs group can be divided into three subtypes: myCAFs, apCAFs, and iCAFs. Created with Figdraw.com.

**Figure 2:**
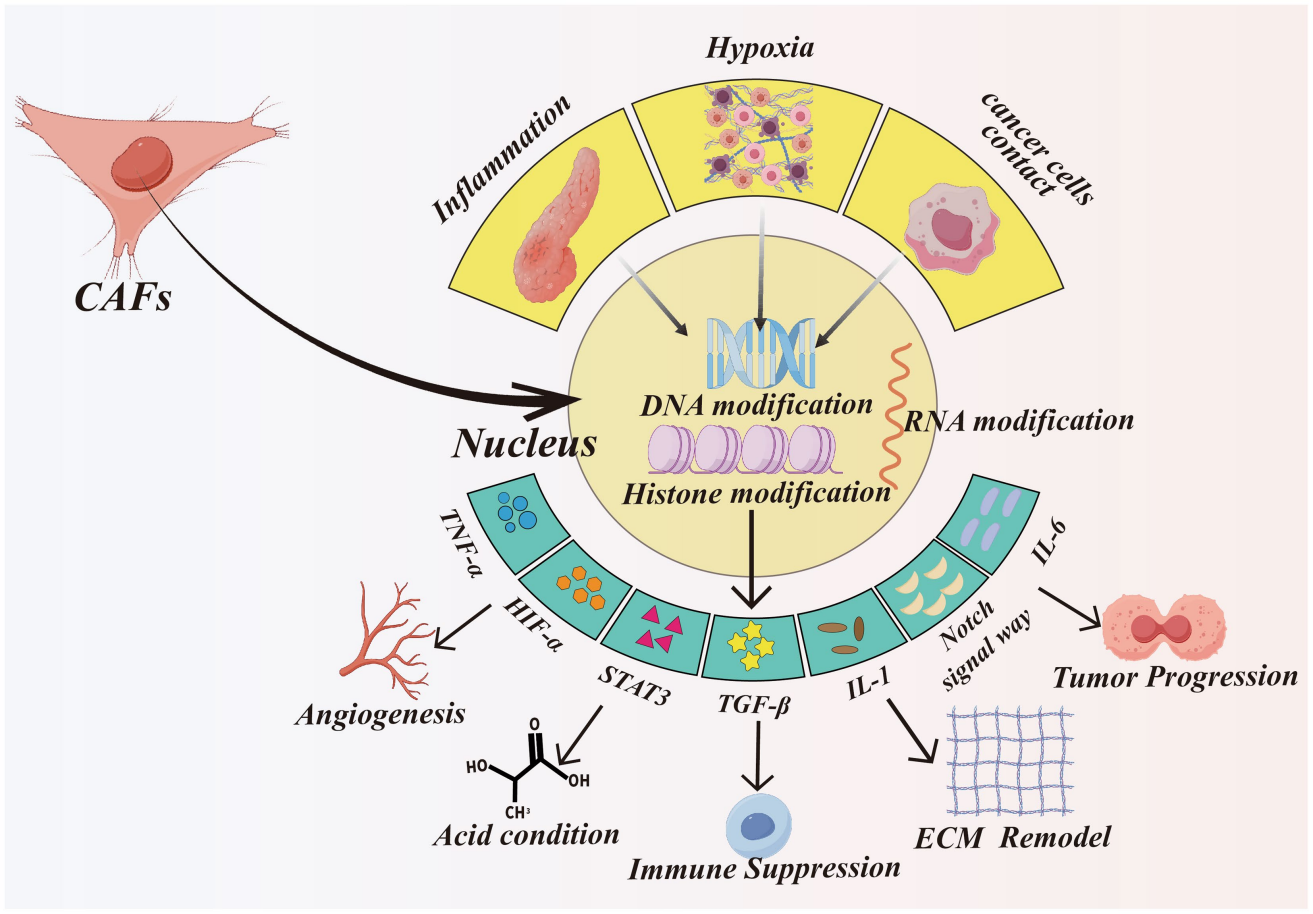
The comprehensive analysis how CAFs epigenetic modification influence tumor progression. The factors inducing epigenetic modification in CAFs include: inflammation, hypoxia, and cancer cells contact and so on. The forms of modification include DNA, RNA, histone modification. CAFs undergoing modification produce many signal factors, such as IL-1, IL-6, TGF-β, TNF-α and so on. These factors will get involved in many biological activities, including produce more acid, remodeling ECM, immune suppression and so on. Created with Figdraw.com.

Recent studies have also suggested that cancer stem cells (CSCs) may contribute to the CAF population, indicating that tumors can reprogram their own stem-like cells into stromal fibroblasts (13). CAFs exhibit substantial functional heterogeneity for cellular diversity, which contributes to the complexity of tumor–stromal interactions (see Figures 1, 2).

After introducing CAF’s origination and substyles, following content will illustrate what epigenetic modification arises in CAFs and the mechanism how these alterations affects CAFs biological action.

### DNA methylation in CAFs

**Figure 3:**
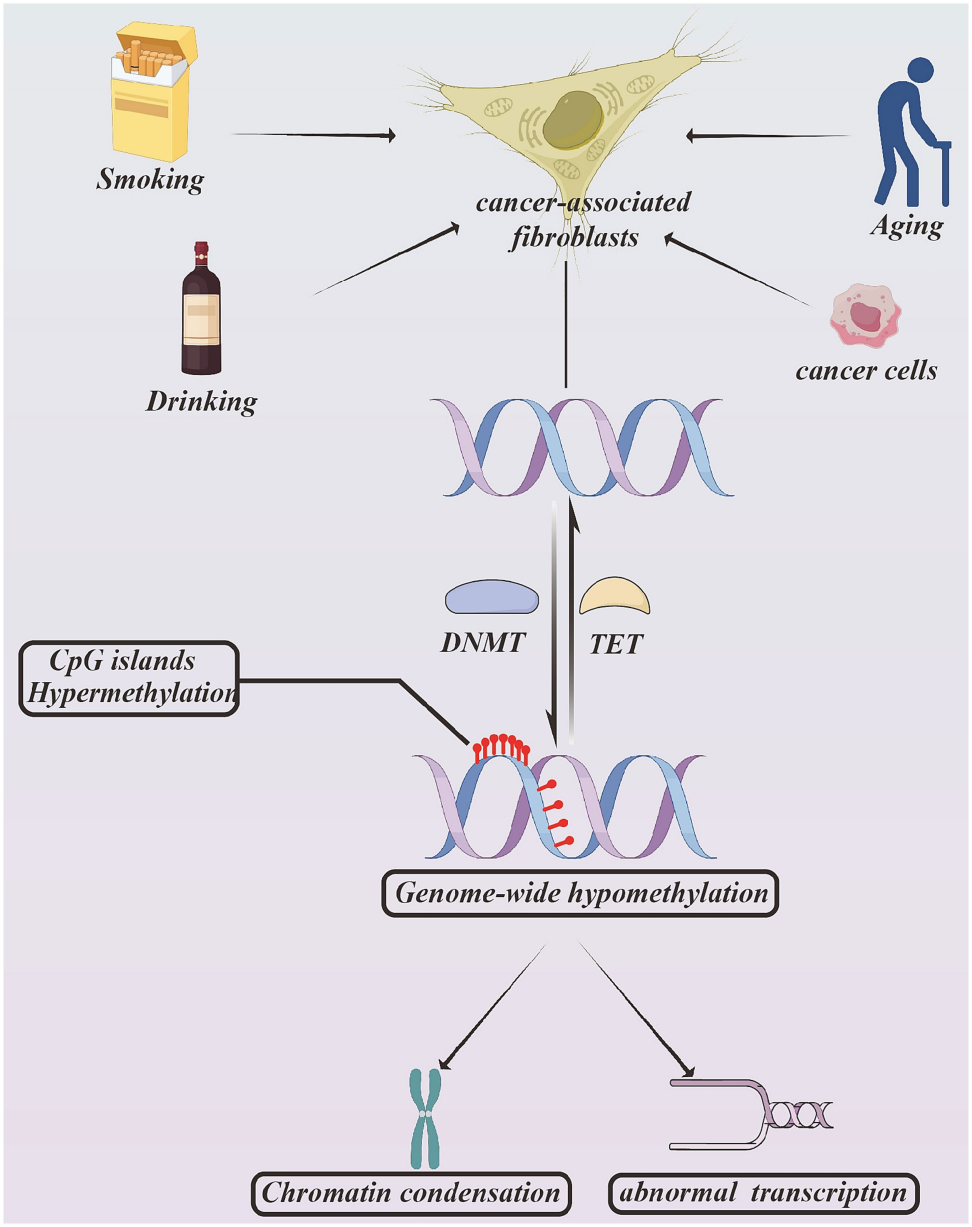
The process and outcome of DNA methylation. The factors influencing DNA methylation modification include smoking, drinking, cancer cells, and aging. This is a reversible process, which DNMT accounts for catalyzing DNA methylation and TET catalyzes adverse process. After modifying, the CpG islands present hypermethylation and whole genome present hypomethylation. This modification result in chromatin construction alteration and abnormal transcription. Created with Figdraw.com.

DNA methylation is a covalent epigenetic modification that occurs at cytosine residues within CpG islands locating in gene promoter regions (14). Hypermethylation of promoter CpG islands typically results in chromatin condensation and transcriptional repression. DNA methylation patterns are established and maintained by DNA methyltransferases (DNMTs) (15). Among DNMT family, DNMT1 maintains methylation patterns, DNMT3A and DNMT3B are responsible for establishing *de novo* methylation (16) (see Figure 3).

Although DNA methylation is generally considered a stable epigenetic modification, it keep in dynamic balance between methylation and demethylation. Demethylation.

has two patterns: one is passive demethylation arising during DNA replication when methylation is not maintained, another one is active demethylation catalyzed by ten–eleven translocation (TET) family of enzymes (17, 18). In general, promoter hypermethylation is associated with gene silencing and hypomethylation is linked to transcriptional activation (19).

The one dominant character of DNA modification is the coexistence of genome-wide hypomethylation and promoter-specific hypermethylation. Environmental factors such as smoking, alcohol, and aging can also induce DNA methylation (20–24). Genome-wide hypomethylation may contribute to genomic instability, promoter hypermethylation frequently silences tumor suppressor genes involved in cell cycle control, apoptosis, DNA repair, and angiogenesis (25). DNMTs are often overexpressed in various cancers and are associated with poor clinical prognosis (26). DNA methylation patterns have been detected in multiple malignant tumor, including colorectal cancer, breast cancer, gastric cancer, and lung adenocarcinomas (27–29).

Compared with normal fibroblasts (NFs), cancer-associated fibroblasts (CAFs) exhibit extensive methylation alterations. For instance, one study identified 1,772 differentially methylated CpG sites between CAFs and NFs, with approximately 60% showing hypermethylation in colon cancer (17). However, other studies report this pattern is not dominant in prostate cancer (30). It suggests DNA methylation is one of mechanisms influencing tumor progression.

DNA methylation is involved in fibroblast activation during fibrotic diseases. DNMT1-mediated methylation induces activation of hepatic stellate cells and contributes to liver fibrosis (2). The promoter 5′-UTR, and gene body of PPARγ contain numerous CpG islands, suggesting potential regulatory methylation sites. PPARγ interacts with the TGF-β/SMAD signaling pathway, playing a key role in fibrosis (31). Another evidence is TGF-β stimulation has been shown to reduce global DNA methylation levels in pulmonary fibrosis (32). Tumor cells can also secrete TGF-β and CXCL12, which activate CXCR4 signaling and stabilize the SMAD-dependent TGF-β pathway, thereby educating the differentiation of normal fibroblasts into CAFs (33).

In pancreatic ductal adenocarcinoma (PDAC), interactions between tumor cells and CAFs induce methylation of the SOCS1 promoter, leading to its downregulation combining with activating STAT3 signaling and promoting tumor progression (34). Additionally, the tumor microenvironment often keeps in high levels of lactic acid condition, which induces demethylation of CXCR4 and increases levels of 5-hydroxymethylcytosine (5hmC), ultimately enhancing CAF-mediated chemokine production and tumor invasiveness (34–36). And DNA methylation also regulates the expression of extracellular matrix–related genes, including COL1A1, COL1A2, and components of the TGF-β/SMAD signaling pathway (37, 38).

Notably, cancer cells can secrete TGF-β, thereby inducing the transformation of NFs into CAFs. During this process, CAFs undergo DNA methylation changes that subsequently enhance TGF-β transcription, forming a positive feedback loop between cancer cells and CAFs.

### Epigenetic modifications of histone in CAFs

Nuclear DNA and histone organize into nucleosomes which is a highly ordered structure. The histone forms the core structural unit of chromatin, which consists of four types of core histone proteins with two copies of each type, including H2A, H2B, H3, and H4. Approximately 147 base pairs of DNA are wrapped around this histone complex (39, 40). Histones undergo extensive post-translational covalent modifications. Acetylation, methylation, and phosphorylation dynamically remodel chromatin architecture by loosening or tightening chromatin, regulating transcription factors, and recruiting regulatory proteins that regulate gene expression (41–43). These modifications alter histone charges, disrupting electrostatic interactions with DNA, and engage in complex crosstalk, collectively influencing transcriptional programs (30, 44).

### Histone acetylation in CAFs

Histone acetylation is a reversible process that is catalyzed by histone acetyltransferases (HATs), removed by histone deacetylases (HDACs), and interpreted by bromodomain and extraterminal (BET) proteins that recognize acetylated histone marks (45). The major HAT families include p300/CBP, GNAT, MYST, p160, PCAF, and TAFII230 (46). HDACs are classified into two main groups: Zn^2+^-dependent deacetylases and NAD^+^-dependent sirtuins. The BET family consists of four members—BRD2, BRD3, BRD4, and BRDT. Among them, BRD4 can specifically binds to acetylated histones and promotes transcriptional activation (47).

The dynamic balance between histone acetylation and deacetylation in CAFs plays a critical role in cancer development and progression. The reader protein BRD4 is upregulated in advanced pancreatic cancer and correlates with increased enrichment of H3K27ac (48). In gastric cancer, BRD4 and H3K27ac co-occupy the enhancer region of *SAA1*, thereby promoting CAF activation and metastatic potential; pharmacological inhibition of BRD4 can reverse this phenotype (49). Similarly, in breast cancer-associated CAFs, EP300-mediated H3K27 acetylation upregulates genes involved in collagen synthesis (50).

**Figure 4:**
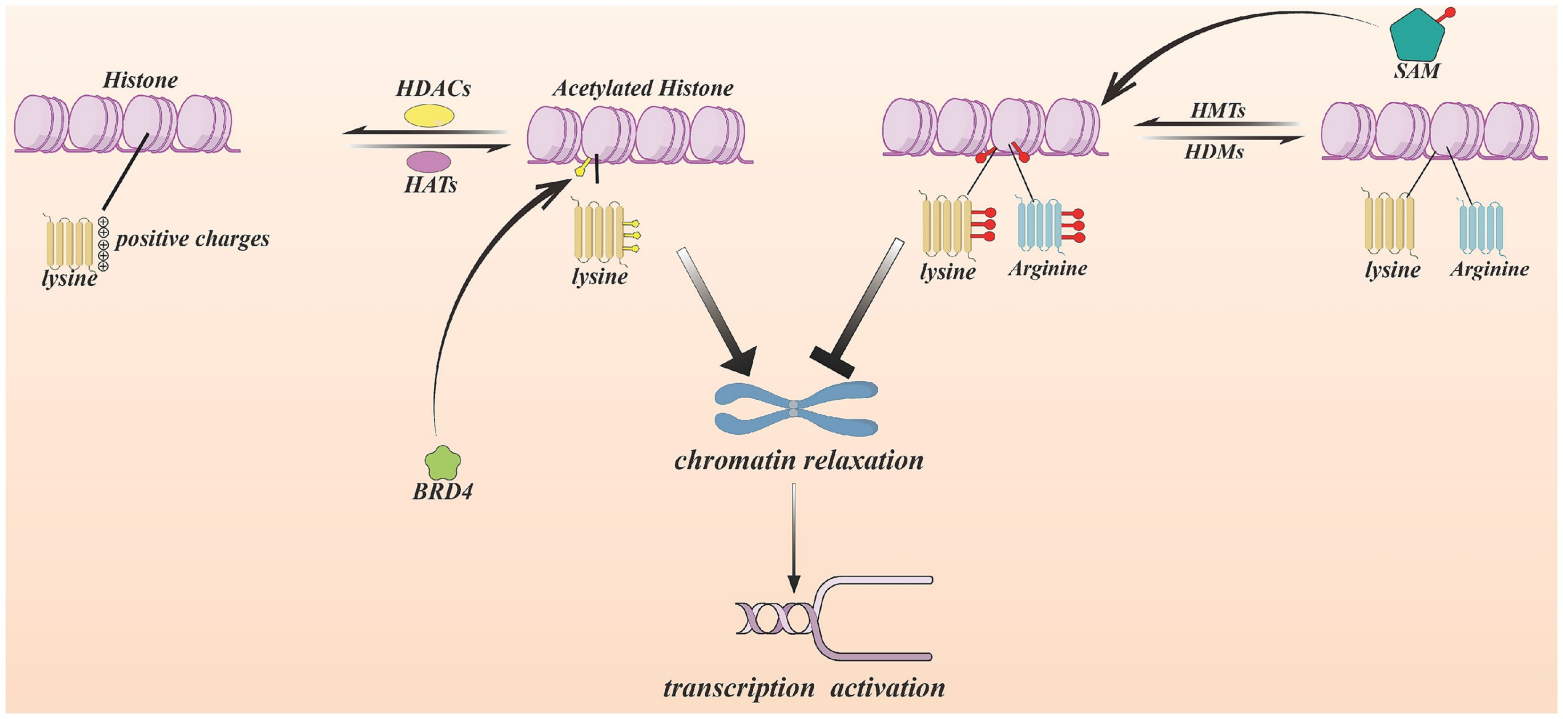
How histone modification effect CAFs. The histone modification in this figure include two sections. One is histone acetylation, another one is methylation. Histone acetylation occurs in histone lysine residues by HATs, it neutralizes positive charges. The adverse reaction is catalyzed by HDACs. The histone acetylation relax chromatin structure, which contributing to genetic transcription. BRD4 recognize acetylated histone and bind it, following promoting transcription. Created with Figdraw.com.

Acetylation of lysine residues neutralizes their positive charge, leading to chromatin relaxation and enhanced accessibility of DNA to the transcription. In contrast, HDACs remove acetyl groups from histones, promoting chromatin condensation and transcription repression. Increasing evidence suggests that HDACs play complex roles in fibrosis: some HDACs promote fibrotic processes (51). However, HDACs do not uniformly promote tumor progression. A study find different HDAC inhibitors may result in opposite effects within pancreatic ductal adenocarcinoma (PDAC) mouse models. Certain HDAC family members are associated with immunosuppression, and inhibition of these HDACs can alleviate immunosuppressive conditions and enhance T-cell activity (46) (see Figure 4).

### Histone methylation

Histone methylation is mediated by two major enzyme families: histone methyltransferases (HMTs) and histone demethylases (HDMs). HMTs catalyze the transfer of methyl groups from S-adenosylmethionine (SAM) to histones and HDMs remove these modifications. Histone methylation predominantly occurs in lysine and arginine residues located on the N-terminal tails of histones (52). Unlike acetylation, histone methylation does not alter histone charges (53).

Histone lysine methyltransferases (HMTs) catalyze lysine methylation, histone lysine demethylases function as “erasers” that remove methyl groups (54). Arginine methylation is mediated by protein arginine methyltransferases (PRMTs), including PRMT1, PRMT3, PRMT4/CARM1, and PRMT5 (55). In general, Lysine methylation represents a relatively stable and complex epigenetic modification occuring on histones H3 and H4. Histone H3 contains several lysine residues that can undergo methylation. H3K4 and H3K36 methylation are generally associated with transcription activation, whereas H3K9, H3K27, and H3K20 methylation are typically linked to transcription repression. In contrast, H3K79 methylation is associated with transcription activation (52).

Methylated histones can regulate the expression of inflammatory mediators and activate signaling pathways. Nuclear factor-κB (NF-κB) is a classical mediator of inflammatory responses, and histone methylation plays an important role in NF-κB-dependent inflammation (56). Histone methylation can also regulate fibrosis-related genes, thereby influencing extracellular matrix accumulation (57). Transforming growth factor-β1 (TGF-β1) is a well-recognized pathogenic factor in fibrosis across multiple organs (58). The TGF-β1/Snail1 axis can recruit PRMT1 and PRMT4 to promote arginine methylation, following driving CAF activation and fibronectin production.

Among histone methylation marks, H3K27 methylation is one of the most extensively studied modifications and is predominantly enriched in promoter and enhancer regions. This modification generally represses transcription and participates in cellular differentiation and cancer progression. The methylation status of H3K27 is dynamically regulated by the opposing activities of HMTs and HDMs. These epigenetic modification are closely associated with chromatin structure, cellular function, inflammation, and tumor progression (59). Specifically, the histone methyltransferase enhancer of zeste homolog 2 (EZH2) mediates H3K27 methylation, leading to chromatin compaction and transcriptional silencing, thereby influencing inflammation and cancer progression (60).

EZH2 is a critical regulator of cancer progression. The tumor microenvironment frequently exhibits hypoxic conditions, which induce the upregulation of hypoxia-inducible factor-α (HIF-α) (61). EZH2 expression can be regulated by HIF-α and contributes to tumor angiogenesis, metastasis, and invasion. To maintain active differentiation states, DNA repair capacity, and cellular plasticity, histones often undergo demethylation. The key enzymes responsible for this process include JMJD3 and UTX, which convert H3K27me2 and H3K27me3 to monomethylated H3K27 (H3K27me1). In actively transcribed genes, enrichment of H3K27me1 antagonizes EZH2-mediated transcriptional repression, thereby influencing cellular fate decisions (62).

**Figure 5:**
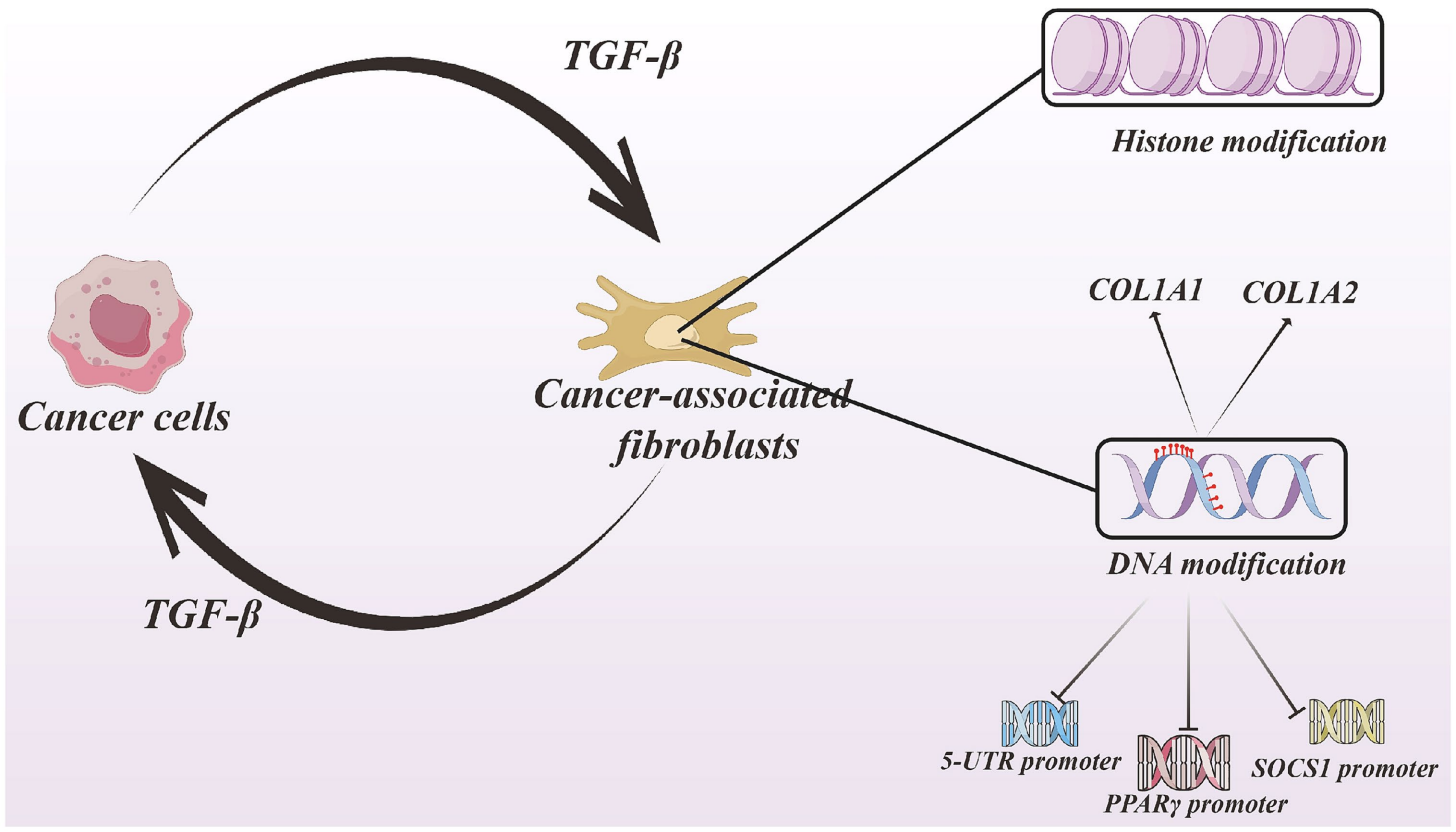
The positive feedback loop of TGF-β. Cancer cells secrete TGF-β factor, which can make CAFs occur DNA and histone modification. The modification promoting CAFs producing this factor combining with the upregulation of COL1A1 and COL1A2. Derived-CAFs TGF-β promote tumor cells progressing. And DNA modification can repress the expression of 5-UTR, PPARγ, SOCS1. Created with Figdraw.com.

In addition, upregulation of nicotinamide N-methyltransferase (NNMT) drives CAFs to produce biomarkers, cytokines, and pro-tumorigenic extracellular matrix components. Depletion of NNMT increases histone methylation marks such as H3K4me3 and H3K27me3, whereas NNMT overexpression decreases these methylation marks at target gene (15) (see Figure 5).

### RNA modifications in CAFs

RNA molecules function not only as targets but also as key mediators of epigenetic regulation, including messenger RNA (mRNA), microRNA (miRNA), and long non-coding RNA (lncRNA). To date, more than 150 RNA chemical modifications have been identified, among which N^6^-methyladenosine (m^6^A), 5-methylcytosine (m^5^C), and N^7^-methylguanosine (m^7^G) are the most extensively studied (63, 64). These modifications regulate RNA stability, splicing, localization, and translation efficiency, thereby influencing gene expression and cellular functions.

Increasing evidence indicates that RNA epigenetic modifications play critical roles in tumor progression and in regulating the phenotypes of cancer-associated fibroblasts (CAFs). Bi et al. (65) reported that *Helicobacter pylori*-induced hypermethylation of miR-149 in CAFs enhances IL-6 secretion through activation of the COX-2/PGE₂ signaling pathway, promoting epithelial–mesenchymal transition (EMT) and stem-like characteristics in gastric cancer cells.

**Figure 6:**
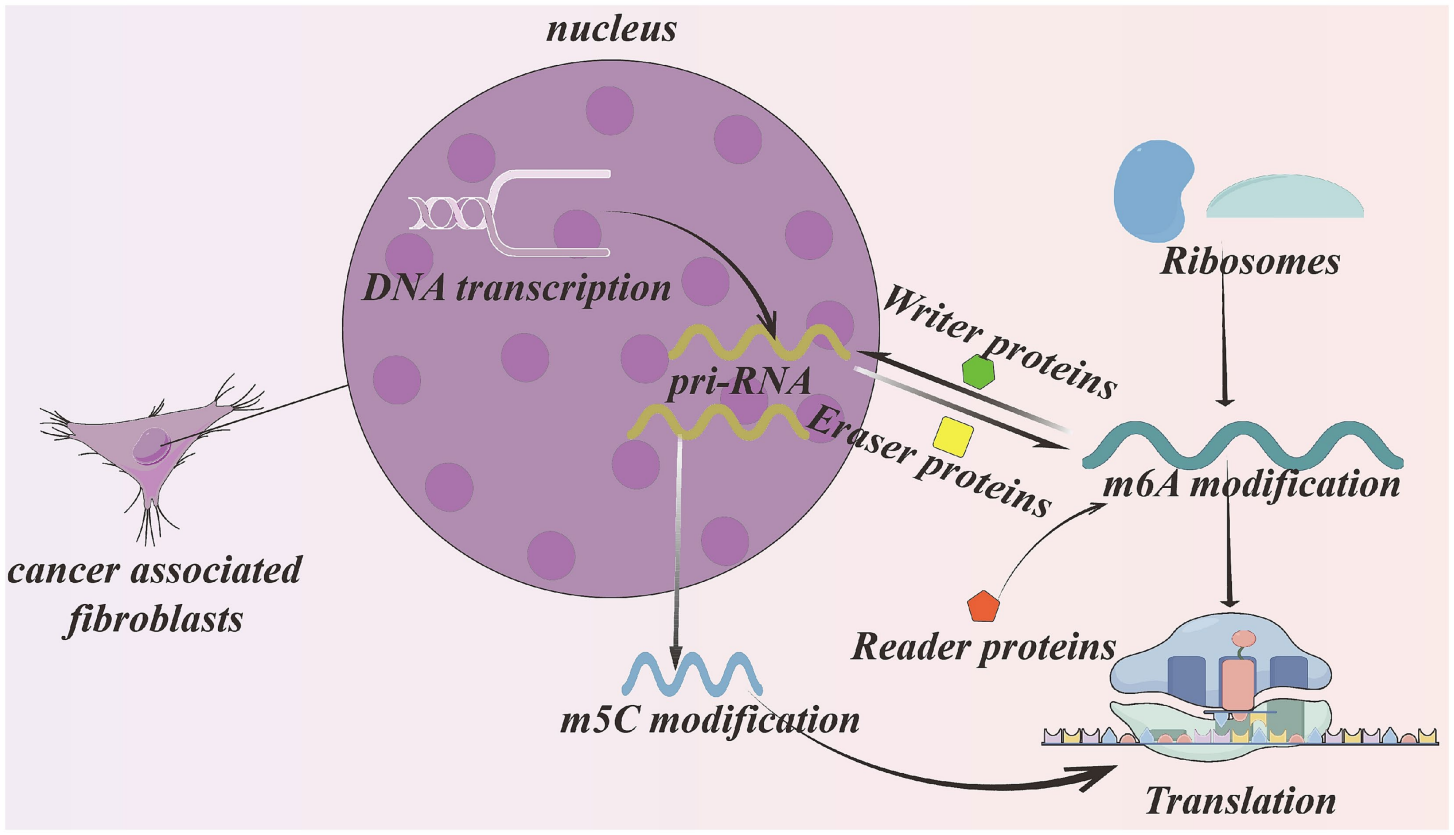
The m^6^A modification and m^5^C modification in CAFs. Two modifications can promote pri-RNA exporting from nucleus. The enzymes group accounting for m^6^A modification consist of writer proteins, reader proteins, and eraser proteins. The m^6^A modification and m^5^C modification can stabilize RNA structure and protect them from degradation and promote translation. The m^6^A modification can also recruit ribosomes to induce translation. Created with Figdraw.com.

Among the various RNA modifications, m^6^A and m^5^C have attracted particular attention because of their significant roles in regulating CAF activation and tumor-promoting functions (see Figure 6).

### N^6^-methyladenosine (m^6^A) in CAFs

m^6^A represents the most prevalent internal modification in eukaryotic mRNAs and plays a critical role in regulating RNA splicing, half-life, and translation efficiency. This modification is installed by the METTL3–METTL14 methyltransferase complex, removed by the demethylases FTO and ALKBH5, and recognized primarily by YTHDF family reader proteins (66, 67).

The m^6^A modification is dynamically regulated by three groups of proteins:

- Writers: methyltransferases such as METTL3 and METTL14 that install m^6^A marks.
- Erasers: demethylases such as FTO and ALKBH5 that remove m^6^A modifications.
-Readers: RNA-binding proteins, including YTHDF family members, that recognize m^6^A-modified transcripts and mediate downstream biological effects (68).

Through the coordinated actions of these regulatory proteins, m^6^A modification influences multiple aspects of RNA metabolism, including RNA stability, splicing, nuclear export, translation efficiency, and degradation (69). Following methylation, mRNAs are recognized by m^6^A-binding proteins that interact with RNA-processing factors, following altering RNA secondary structures and influencing splicing patterns. These modifications can also facilitate nuclear export by recruiting export receptors and enhance translation by promoting ribosome recruitment (70). In addition, m^6^A modification regulates microRNA processing by recruiting DGCR8 to facilitate the maturation of primary miRNAs (pri-miRNAs) (71, 72).

Recent studies have demonstrated that m^6^A RNA methylation participates in fibroblast activation and fibrotic diseases. The m^6^A modification has been shown to regulate the activation of hepatic stellate cells, contributing to liver fibrosis (2). In this process, METTL3 and METTL14 increase m^6^A modification within the 5′-UTR of TGF-β mRNA, while the reader protein YTHDF1 enhances TGF-β translation efficiency, thereby promoting fibroblast activation.

Furthermore, m^6^A modification is closely associated with metabolic reprogramming in cancer. METTL3-mediated m^6^A modification can increase the expression of glycolytic enzymes and enhance aerobic glycolysis in tumor cells (68). Under hypoxic conditions in the tumor microenvironment, the levels of HIF-1α increase and participate in promoting tumor development. METTL3-induced m^6^A modification further enhances HIF-1α expression and promotes glycolytic metabolism.

The m^6^A-dependent regulation of glycolytic enzyme transcripts has been reported in numerous malignancies, including colon, lung, pancreatic, renal, cervical, and gastric cancers, as well as osteosarcoma (73–82). Lactic acid–mediated histone H3K18 lactylation has been shown to regulate m^6^A modification of downstream targets by increasing the transcription of related enzymes (83). In addition, metabolic by-products in the tumor microenvironment may influence epigenetic modifications. Lactic acid–mediated histone lactylation at H3K18 can increase the transcription of m^6^A-related enzymes, thereby regulating downstream RNA methylation processes (84).

**Figure 7:**
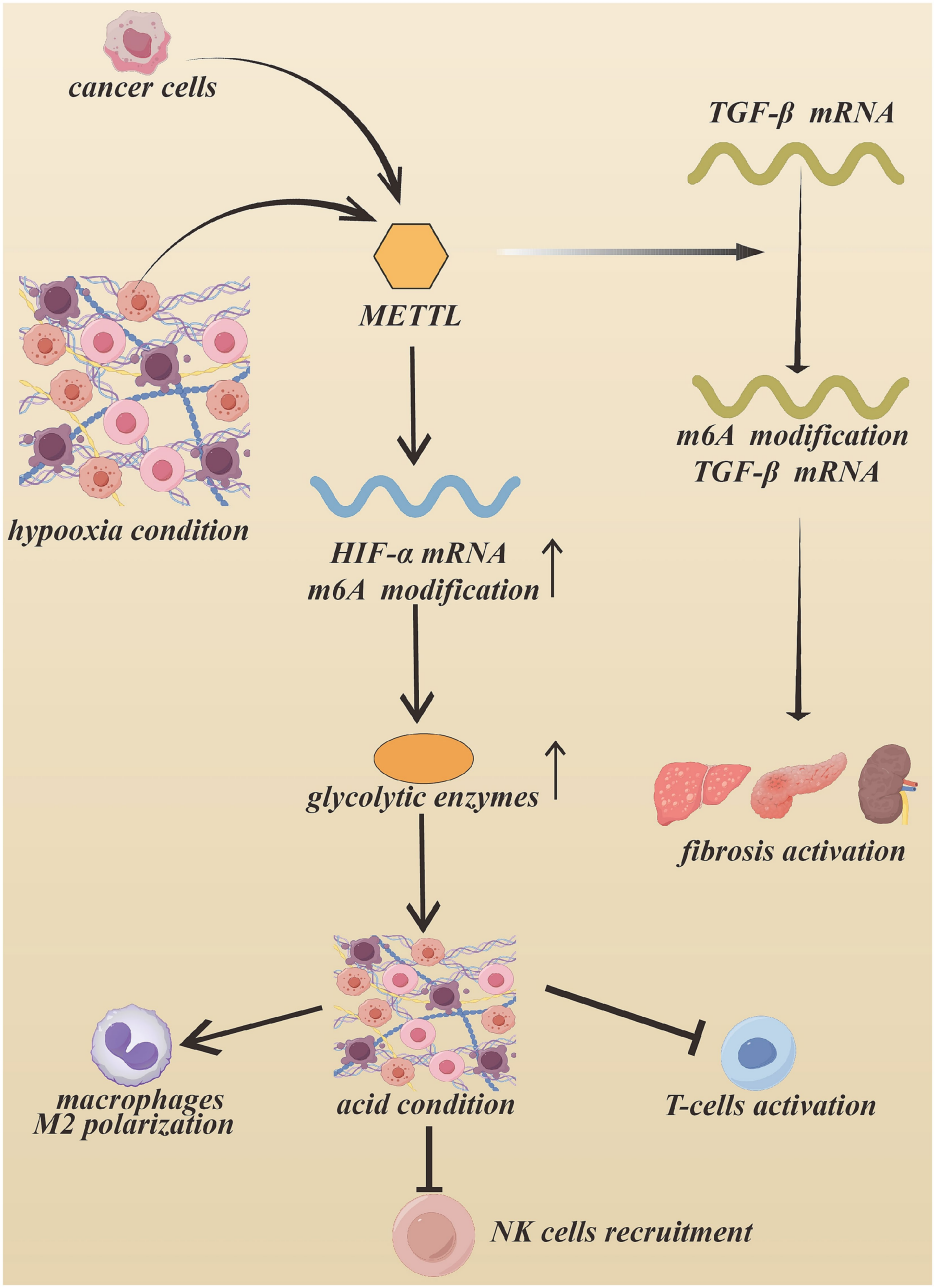
METTL family m^6^A modification METTL family can stabilize TGF-β mRNA, following activating fibrotic genes and induce fibrosis. In addition, tumor microenvironment keeps in a hypoxia condition, which induces CAFs enhance HIF-α level. The upregulation of HIF-α promote the synthesis of glycolytic enzymes, which accelerate glucose consumption and producing more acid. The acid environment suppress the recruitment of immune cells, such as NK cells and T cells. Created with Figdraw.com.

Within the tumor microenvironment, elevated glucose consumption leads to increased lactic acid production, creating an acidic environment that contributes to immune suppression. Under these conditions, macrophages frequently undergo M2 polarization, while the survival and activity of T cells and natural killer (NK) cells are suppressed. For instance, m^6^A-mediated stabilization of circQSOX1 has been reported to facilitate lactic acid accumulation and promote the recruitment of regulatory T cells (Tregs), thereby contributing to tumor immune evasion (85) (see Figure 7).

### m^5^C RNA modification

The m^5^C RNA modification is widely distributed across multiple RNA species, including tRNA, rRNA, mRNA, and non-coding RNAs. Among these RNA types, tRNA and rRNA exhibit the high abundance of m^5^C modifications.

The enzymes for catalyzing m^5^C modifications mainly include members of the NOL1/NOP2/SUN (NSUN) family and tRNA aspartic acid methyltransferase 1 (TRDMT1). Different enzymes display substrate specificity: NSUN2 and NSUN4 modify mRNA, tRNA, and miRNA. NSUN6 and TRDMT1 mainly target tRNA. NSUN1 and NSUN5 primarily modify rRNA among these enzymes, NSUN2 plays a particularly important role in regulating m^5^C levels in mRNA (63).

Unlike DNA methylation and m^6^A RNA methylation, the existence of specific m^5^C demethylases (“erasers”) remains uncertain (86). However, m^5^C can undergo stepwise oxidative modification rather than direct demethylation. This process is catalyzed by enzymes such as ALKBH1 and the ten–eleven translocation (TET) family, generating the stable intermediates 5-hydroxymethylcytosine (hm^5^C), 5-formylcytosine (f^5^C), and 5-carboxylcytosine (ca^5^C) (87). These oxidative derivatives may influence RNA structure and function.

m^5^C modification plays essential roles in maintaining RNA stability and regulating translation. The m^5^C modification can stabilize RNA secondary structures and influence the stem–loop of tRNA molecules (88). In rRNA, m^5^C modification at position C2278 in 25S rRNA contributes to the stabilization of ribosome structure. Additionally, hypermethylated mRNAs can be stabilized through YBX1-dependent mechanisms (86, 89). NSUN2-mediated m^5^C modification also regulates the processing of vault RNA, facilitating its conversion into miRNA and protecting it from degradation (68, 87, 90).

Furthermore, m^5^C modifications can regulate mRNA nuclear export. The m^5^C reader protein ALYREF recognizes methylated RNA sequences and promotes the export of mRNA from the nucleus to the cytoplasm (91). In bladder cancer, ALYREF binding to the 3′-UTR of PKM2 mRNA stabilizes the transcript and promotes glycolysis and tumor growth (91).

That m^5^C RNA modification plays crucial roles in RNA stability, nuclear export, and translational regulation, following regulating various cellular fates. NSUN2-induced m5C methylation of the CNTTB1 mRNA can influence uveal melanoma cell proliferation and migration through blocking cycle G1 stage (92). The upregulation of m5C modified lncRNA NMR are associated with drug resistance, which may relate to the expression of MMP3 and MMP10. Undergoing m5C modification, epithelial differentiation is inhabited in pancreatic cancer (93).

In addition, the removal of m5C modification also regulate the stability of RNA.

**Figure 8:**
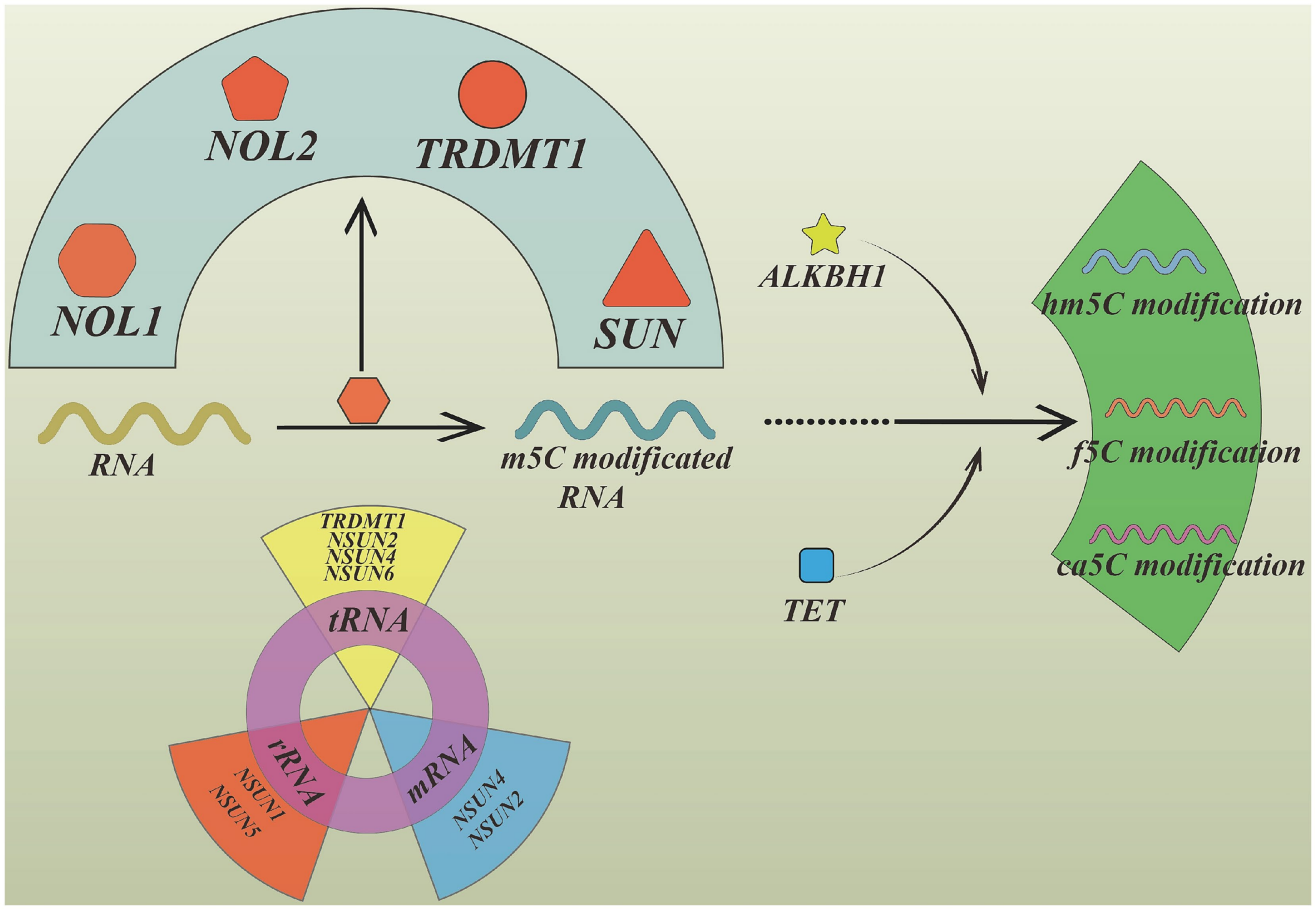
The m^5^C modification. The enzymes catalyzing RNA m^5^C modification mainly include NOL1, NOL2, TRDMT1, SUN family. Among these enzymes, SUN family’s functions are complex and different numbers are responsible for catalyzing different RNA. NSUN2, NSUN4, NSUN6, TRDMT1 mainly catalyzing tRNA, NSUN1, NSUN5 mainly catalyzing rRNA, NSUN2, NSUN4 mainly catalyzing mRNA. The removal of m5C modification is not a directional process, which is transformed into hm5C RNA, f5C RNA, ca5C RNA through catalyzed by TET and ALKBH. Created with Figdraw.com.

The decrease of TET2 accelerate m5C accumulation in TSPAN13 mRNA, YBX1 specially recognize m5C sites and increase the expression of TSPAN13 transcription in acute myeloid leukemia (94) (see Figure 8).

## Discussion

### Toward an epigenetic re-education of CAFs in cancer therapy

The study of epigenetic regulation in cancer-associated fibroblasts (CAFs) extends beyond merely cataloging epigenetic modifications; it prompts a fundamental reconsideration of stromal reprogramming in cancer therapy. In addition to elucidating how DNA methylation, histone modifications, and RNA methylation contribute to CAF activation, emerging evidence highlights the potential of an “epigenetic re-education” strategy, whereby tumor-supportive fibroblasts are reprogrammed into quiescent or even tumor-restraining stromal cells.

### Beyond genetic determinism: the plastic CAF epigenome

The CAF epigenome represents a dynamic interface between intrinsic genetic programming and extrinsic environmental cues. In contrast to the relative stability of genetic mutations, epigenetic modifications in CAFs exhibit remarkable plasticity and can rapidly respond to metabolic stress, immune signaling, and therapeutic pressures within the tumor microenvironment. Although such plasticity complicates therapeutic targeting, it also positions CAFs as critical sensors and integrators of microenvironmental stress signals. Importantly, recent studies suggest that epigenetic therapeutics may not only inhibit CAF activity but also actively reprogram CAF function, indicating a conceptual shift from simply targeting stromal cells to reprogramming stromal phenotypes.

### Therapeutic paradoxes and context dependence

The multifaceted roles of CAF epigenetic regulation give rise to several therapeutic paradoxes that warrant careful consideration. For example, although inhibition of histone deacetylases (HDACs) is generally associated with suppression of tumor growth, such inhibition may paradoxically activate inflammatory CAF subsets under certain conditions. Similarly, modulation of N^6^-methyladenosine (m^6^A) signaling can exert context-dependent effects across different cancer types, promoting tumor progression in colorectal cancer while enhancing antitumor immune responses in non-small cell lung cancer. These context-dependent outcomes highlight the importance of biomarker-guided therapeutic strategies that account for tumor-specific CAF ecosystems rather than relying on uniform epigenetic interventions.

### The spatial dimension of CAF epigenetics

Recent advances in spatial transcriptomics and epigenomics have revealed substantial heterogeneity in CAF epigenetic states across different spatial regions of tumors. CAF subpopulations located near invasive fronts, vascular niches, or immune-cell–rich regions may exhibit distinct epigenetic signatures and functional phenotypes. Such spatially regulated epigenetic heterogeneity presents challenges for therapeutic delivery but simultaneously offers opportunities for spatially informed combinational therapies that selectively target specific stromal niches.

### Toward next-generation CAF-directed epigenetic therapies

Future CAF epigenetic targeting requires several paradigm shifts:

1 Temporal precision: recognizing that epigenetic interventions may have opposing effects at different disease stages.
2 Spatial consideration: accounting for the topographic organization of CAF subtypes.
3 Combinatorial intelligence: designing epigenetic combinations that simultaneously target multiple CAF subpopulations.
4 Dynamic monitoring: developing functional epigenetic biomarkers to track CAF reprogramming in real time.

The recent failure of broad epigenetic modulators in stroma-rich cancers highlights the limitations of non-selective approaches. Instead, the future lies in developing CAF subtype-specific epigenetic drugs that can precisely modulate distinct aspects of the CAF phenotype—whether immunomodulatory, matrix-remodeling, or metabolic functions.

### The stromal epigenome as a therapeutic Interface

The CAF epigenome represents more than a collection of molecular modifications; rather, it constitutes a dynamic therapeutic interface linking cancer cells with their surrounding microenvironment. Recognizing CAF epigenetic states as plastic, responsive, and potentially reversible opens new opportunities for stromal normalization strategies that may complement existing anticancer therapies. Moving forward, the central challenge will not simply be the identification of additional epigenetic modifications but rather the development of strategies capable of orchestrating these regulatory layers to transform the pro-tumorigenic CAF program into a therapeutic ally.

## Conclusion

DNA methylation, histone modifications, and RNA methylation operate in a coordinated manner to drive the activation of cancer-associated fibroblasts (CAFs) into a tumor-supportive state and to fine-tune their functional roles within the tumor microenvironment. Accumulating evidence across multiple malignancies highlights CAFs as attractive therapeutic targets, largely owing to their pronounced epigenetic plasticity and relative genomic stability compared with cancer cells. Nevertheless, the clinical translation of CAF-directed epigenetic therapies remains challenging. Future research should therefore prioritize subtype-specific epigenetic modulation, strategies targeting stromal–tumor cell crosstalk, and biomarker-guided patient stratification to fully harness the therapeutic potential of CAF reprogramming in cancer treatment.
